# Methadone-involved overdose deaths in urban and rural communities before and after the public health emergency flexibilities for methadone take-home doses

**DOI:** 10.1016/j.dadr.2025.100339

**Published:** 2025-04-24

**Authors:** Rebecca Arden Harris, Judith A. Long, Yuhua Bao, Henry R. Kranzler, Jeanmarie Perrone, David S. Mandell

**Affiliations:** a Department of Family Medicine and Community Health, Perelman School of Medicine, University of Pennsylvania, Philadelphia, PA, USA; b Leonard Davis Institute of Health Economics, University of Pennsylvania, Philadelphia, PA, USA; c Corporal Michael J. Crescenz VA Medical Center, Philadelphia, PA, USA; d Division of General Internal Medicine, Perelman School of Medicine, University of Pennsylvania, Philadelphia, USA; e Department of Population Health Sciences, Weill Cornell Medicine, New York, NY 10065, USA; f Department of Psychiatry, Perelman School of Medicine, University of Pennsylvania, Philadelphia, PA 19104, USA; g Department of Emergency Medicine, Perelman School of Medicine, University of Pennsylvania, Philadelphia, PA, USA; h Center for Addiction Medicine and Policy, University of Pennsylvania, Philadelphia, PA 19104, USA

**Keywords:** Methadone, Opioid treatment program, Overdose, Take-home doses, Urban-rural, COVID-19

## Abstract

**Background:**

To mitigate COVID-19 exposure risks in methadone clinics, the Substance Abuse and Mental Health Services Administration (SAMHSA) issued a temporary modification of regulations in March 2020 to permit extended take-home methadone doses: up to 28 days of take-home methadone for stable patients and 14 days for those less stable. This study examined the association between the policy change and fatal methadone overdoses across the urban-rural continuum.

**Methods:**

This interrupted time series analysis used the U.S. National Vital Statistics System (NVSS) 2018–2022 mortality data to examine monthly trends in methadone-involved overdose deaths before and after the policy change allowing more take-home methadone doses. Deaths were stratified into six urban-rural categories and by co-involvement of fentanyl.

**Results:**

Prior to the policy change, trends in methadone-involved overdose deaths were either flat or declining across all urbanization categories. Following the policy change, deaths decreased significantly in Large Central Metro areas but increased in rural Micropolitan counties. No trend changes occurred in the other urban or rural categories. When stratified by fentanyl co-involvement, Large Central Metro areas experienced a decrease in methadone deaths with fentanyl, though not statistically significant, and a significant decrease without fentanyl. In rural Micropolitan counties, methadone deaths saw an increase with fentanyl co-involvement that did not reach significance, and a significant increase without fentanyl. Noncore counties saw a significant increase in deaths involving both methadone and fentanyl, with no notable change observed without fentanyl.

**Conclusions:**

Results suggest the need to expand methadone access and treatment supports in underserved rural communities, recognizing that factors beyond the policy change may have contributed to the reported associations.

## Highlights

• In response to the pandemic, SAMHSA eased restrictions on take-home methadone doses.
• We analyzed the impact on methadone overdose deaths across urban-rural areas.
• Large urban areas saw a decline in methadone deaths after take-home policy change.
• Some rural areas saw a rise in methadone deaths after the policy change.
• Findings suggest bolstering methadone support services in rural communities.

## 1 Introduction

Since 1972, when the US Food and Drug Administration approved the use of methadone for treating opioid use disorder, there has been tight governmental control through federal statutes and regulations that allow only federally certified opioid treatment programs (OTPs) to dispense the medication. This restrictive legal framework was designed to meet three objectives: treat individual patients, protect public safety, and bolster public health (Institute of Medicine, 1995). Before the COVID-19 pandemic, most patients receiving methadone had to appear in-person for initial evaluations and then in-person every morning 5–6 days per week for directly observed therapy. On March 16, 2020, in response to the first wave of the pandemic, the Substance Abuse and Mental Health Services Administration (SAMHSA) issued a blanket exception that allowed OTPs, with state concurrence, to provide up to 28 days of take-home methadone for clinically stable patients and 14 days for those who were less stable (Substance Abuse and Mental Health Services Administration, 2022). This exception remained in effect until February 2024, when SAMHSA updated federal regulations to permanently expand take-home dosing flexibilities (U.S. Department of Health and Human Services, 2024). Although research on the effects of the take-home policy is still emerging, published findings suggest that the policy increased the number of patients receiving take-home doses without an adverse effect on outcomes, including fatal overdoses (Adams et al., 2023, Krawczyk et al., 2023, Williams et al., 2023). Stakeholders reported greater engagement with treatment and patient satisfaction, with little misuse or diversion (Dooling and Stanley, 2021, Figgatt et al., 2021, Joseph et al., 2021, Welsh et al., 2022, Fuller et al., 2024).

Despite the federal expansion, states can still choose whether to participate (U.S. Department of Health and Human Services, 2024). Some states, including Arizona, Florida, Indiana, Ohio, and Mississippi, had initially allowed extended take-home privileges during the COVID-19 pandemic but later rescinded these policies (Roy et al., 2024). Even in states that adopted the exemption, individual OTPs had the discretion to discontinue or limit the practice after the pandemic. New York state has seen a trend towards more restrictive dosing practices (Jordan et al., 2024).

Restrictions on methadone dosing may impede patients' adherence and treatment success, especially among rural patients for whom easing take-home restrictions could offer distinct benefits. Research has shown that rural areas have a shortage of OTP facilities (Jehan et al., 2023), resulting in long distances and drive times to an OTP, often as long as 45 minutes one-way (Joudrey et al., 2019). These, along with other transportation obstacles such as higher travel costs, lack of public transportation, unreliable vehicle, and bad weather, can undermine adherence, particularly for patients with demanding work, childcare, or other responsibilities (Hoffman et al., 2022). We anticipated that relaxing restrictions around take-home methadone doses would lower barriers to treatment engagement and adherence to prescribed dosing regimens, potentially reducing the risks of overdose deaths involving methadone. In this study, we examined the association between expanded access to take-home methadone doses and methadone-involved overdose deaths across the urban-rural spectrum.

## 2 Methods

### 2.1 Data source and study population

In this interrupted time series analysis, we used U.S. National Vital Statistics System (NVSS) 2018–2021 final and January–June 2022 provisional mortality data. NVSS data are based on death certificates for U.S. residents.

In addition to the underlying cause of death, a death certificate may list up to 20 contributing causes (Slavova et al., 2019). The International Classification of Diseases, 10th Revision (ICD-10) codes for underlying cause-of-death in drug overdose deaths are X40-X44, X60-X64, X85, and Y10-Y14. Methadone as a contributing cause of death is identified by ICD-10 code T40.3. Methadone-associated deaths often co-involve illegal drugs (Corkery et al., 2004), such as fentanyl, heroin, cocaine, or methamphetamines. With its long half-life, toxicological analysis can detect methadone in urine 2–4 days after ingestion (Center for Substance Abuse Treatment, 2006, Karch and Stephens, 2000, Substance Abuse and Mental Health Services Administration, 2021); however, death certificates do not specify which drug is primarily responsible for the overdose death and which played a role in combination with other drugs (Gill, 2017).

We compiled monthly methadone-involved overdose deaths from January 2018 to June 2022 (54 months; 27 months before the policy change and 27 after), stratifying by the National Center for Health Statistics urban-rural county classification scheme (Ingram and Franco, 2014). Ranging from most urban to most rural, the classification system includes six categories – Large Central Metro, Large Fringe Metro, Medium Metro, Small Metro, Micropolitan, and Noncore – based primarily on metropolitan–nonmetropolitan status and population distribution (supplement Figure S1). The metropolitan counties are densely populated and include large central counties, the fringes of large counties (suburbs), and medium and small counties. Nonmetropolitan counties include micropolitan areas with urban cores of 10,000–49,999 people that serve as regional hubs, along with Noncore areas such as small towns, farmland, and open countryside. The four metropolitan categories are usually grouped as “urban counties,” while the two nonmetropolitan categories are grouped as “rural counties” (Spencer et al., 2022).

### 2.2 Analytic strategy

We used interrupted time series analysis to model trends in monthly overdose deaths (see supplement part A for model specifications). The method constructs a counterfactual – what the trend would look like in the absence of the policy change – which is then compared with the post-intervention actual trend (Bernal et al., 2017). In constructing the counterfactual, the method accounts for discrete changes in mortality levels occurring at the initial point of intervention (Linden, 2015). A significant difference in the slopes before and after the policy change would suggest that the policy change was associated with either an increase or decrease in methadone-related overdose deaths. We anticipated an implementation lag of 4–6 weeks, even for “early adopters” of the policy change (Department of Health and Human Services, 2020, White et al., 2024). Because our postintervention time series ran 27 months, this lag time would not obscure longer-term changes in the trend lines of methadone-involved deaths.

Considering the expanding role of fentanyl in the overdose crisis (Saloner et al., 2023), we also conducted a stratified analysis to control for the co-occurrence of synthetic opioids, mainly fentanyl and its analogs. We sought to determine whether the associations between the take-home policy and fatal methadone-involved overdose were modified when estimated separately for (1) deaths that involved methadone but not fentanyl, and (2) deaths that involved both methadone and fentanyl. We used ICD–10 code T40.4 to identify synthetic opioid−involved deaths. We did not stratify the data further by drug types such as benzodiazepines or stimulants as there would be too few observations per time point for a stable analysis.

We also assessed whether monthly non−methadone-involved overdose deaths could serve as a secular trend comparison. Such a comparison would aid in determining whether a change in the trend line of methadone-involved deaths was associated with the take-home policy change or attributable to other factors affecting trends in drug overdose deaths generally. Non−methadone deaths satisfied the two *a priori* criteria for a secular trend control: (1) a theory−based relationship with methadone deaths (i.e., methadone and non-methadone overdose deaths may be subject to the same broader social forces, although the populations may differ in some respects), and (2) no theory−based relationship with the policy change (i.e., trends in non-methadone overdose deaths are not dependent on a change in the methadone take-home policy). A third well-established criterion is strictly empirical: were methadone and non-methadone overdose deaths correlated before the policy change? If they were not correlated in the pre-intervention period, then non-methadone overdose deaths do not provide a basis for a post-intervention comparison (Bernal et al., 2018) (see supplement part B for model specifications). Recent studies have used non-methadone overdose deaths for secular trend purposes, but without providing empirical justification (Jones et al., 2022, Kleinman and Sanches, 2023).

### 2.3 Statistical analyses

We used totals and percentages to describe overdose mortality by urbanization category. To conduct the interrupted time series analysis, we used Stata module ITSA to estimate for each urbanization category the pre-intervention slope, the change in mortality level occurring at intervention start, the post-intervention slope, and the change in the pre- and post-intervention slopes. While our dataset covered the entire U.S. population from 2018 to 2022 without sampling, we follow usual practice and report confidence intervals and p-values for each of these parameters, using p < 0.05 as a conventional threshold for significance but interpreting p-values more broadly, recognizing that values close to this threshold may still hold meaningful insights. To adjust for autocorrelation, we used the Akaike information criterion (AIC) for the lag order selection. Interrupted time series analysis specifies the maximum lag to be considered in the autocorrelation structure. We used Spearman’s rho to measure the pre-intervention secular trend correlations. All analyses were performed using Stata, version 18.

Because this study used publicly available, de-identified data, it did not require human subjects review, consistent with guidance from the University of Pennsylvania institutional review board. The study followed the Strengthening the Reporting of Observational Studies in Epidemiology (STROBE) reporting guideline (von Elm et al., 2008).

## 3 Results

Table 1 presents the population characteristics of the six categories studied. The Large Central Metro category had the highest population at 86,027,589, while the smallest population was in the Noncore (Nonmetro) category with 17,620,604 individuals. Non-Hispanic Whites comprised the largest ethnic-racial group in all categories, with their percentage increasing generally with rurality. Table 1 also displays the total methadone-related deaths from January 2018 to June 2022, which ranged from 494 in Noncore to 5563 in Large Central Metro. Large Central Metro areas also had the highest percentage of overdose deaths involving both methadone and fentanyl at 51.0 % and Noncore had the lowest at 24.9 %. Fentanyl co-involvement rose across all urbanization categories during the study period (supplement Figure S2).

**Table 1:** Population characteristics and methadone and fentanyl co-involved deaths by 2013 NCHS Urban-Rural. Classification Scheme for Counties, January 2018–June 2022.

**Parameter**	**Large Central Metro**	**Large Fringe Metro**	**Medium Metro**	**Small** **Metro**	**Micropolitan (Nonmetro)**	**Noncore (Nonmetro)**
**# counties (2013)**	68	368	373	358	641	1335
**Population (2020)**	86,027,589	74,550,573	63,172,433	27,920,449	25,542,425	17,620,604
**Black population**	17,033,102	10,274,893	7553,011	2872,082	2112,274	1581,914
**% Black**	19.8 %	13.8 %	12.0 %	10.3 %	8.3 %	9.0 %
**Hispanic population**	26,314,644	11,568,361	11,740,966	3246,367	2554,100	1215,316
**% Hispanic**	30.6 %	15.5 %	18.6 %	11.6 %	10.0 %	6.9 %
**White population**	42,679,843	52,707,319	43,878,456	21,802,000	20,876,051	14,823,374
**% White**	49.6 %	70.7 %	69.5 %	78.1 %	81.7 %	84.1 %
**# drug overdose deaths**^a^	131,985	96,976	93,387	34,706	31,894	19,149
**# all methadone deaths**^a^	5563	3366	3181	1116	875	494
**% in each of the** **county categories**	38.2 %	23.1 %	21.8 %	7.6 %	6.0 %	3.4 %
**# methadone deaths** **before the policy change**	2395	1515	1428	517	419	232
**% before**	43.1 %	45.0 %	44.9 %	46.3 %	47.9 %	47.0 %
**# methadone deaths** **after the policy change**	3168	1851	1753	599	456	262
**% after**	56.9 %	55.0 %	55.1 %	53.7 %	52.1 %	53.0 %
**# methadone deaths involving fentanyl**^a^	2835	1488	1137	319	270	123
**% fentanyl**	51.0 %	44.2 %	35.7 %	28.6 %	30.9 %	24.9 %
**% in each of the** **county categories**	45.9 %	24.1 %	18.4 %	5.2 %	4.4 %	2.0 %
**# involving fentanyl** **before the policy change**	962	568	391	111	100	36
**% before**	33.9 %	38.2 %	34.4 %	34.8 %	37.0 %	29.3 %
**# involving fentanyl** **after the policy change**	1873	920	746	208	170	87
**% after**	66.1 %	61.8 %	50.1 %	65.2 %	63.0 %	70.7 %
**Covid−19 death rates**^b^	97.7	79.9	75.0	78.2	86.5	90.6

NOTES:

a Over the study period (January 2018–June 2022);

b 2020 age-adjusted rates per 100,000 population

### 3.1 Interrupted time series analysis estimates by urbanization category

**Fig. 1:**
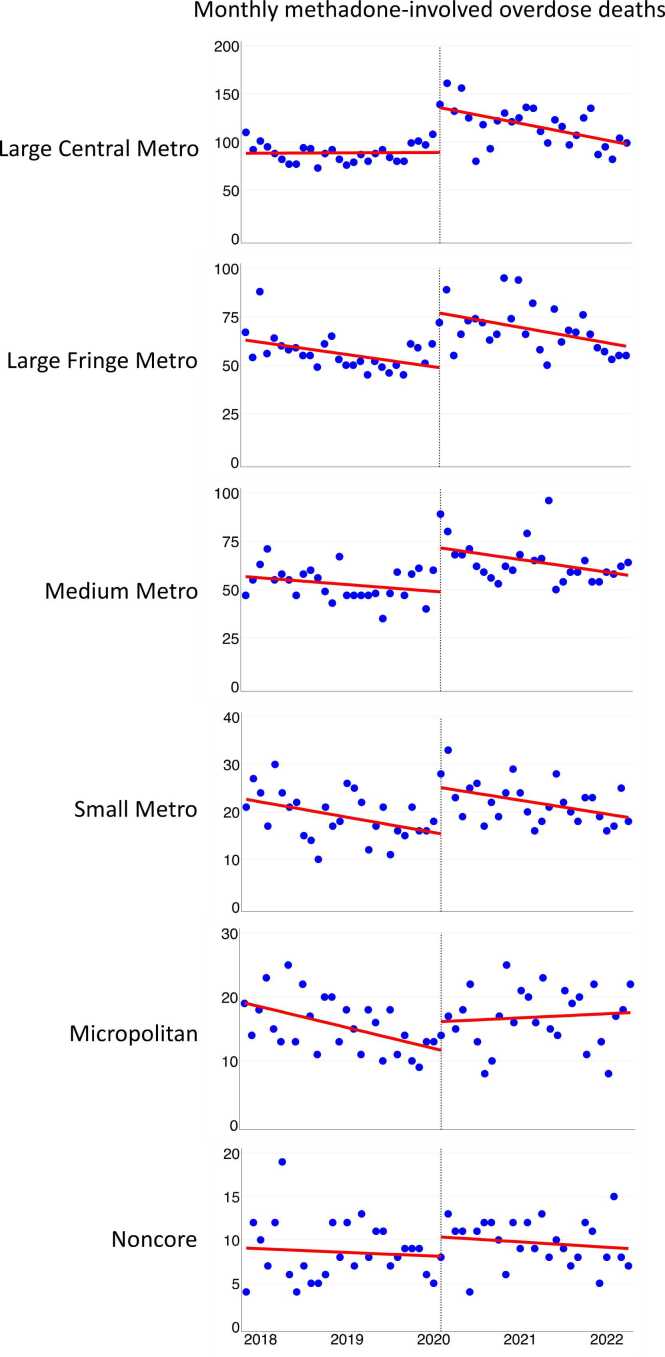
Drug overdose deaths involving methadone by urban–rural categories, January 2018–June 2022.

Source: Authors’ analysis of drug overdose mortality data from the National Vital Statistics System, CDC WONDER online database. NOTES: The circles are actual deaths, trend lines are predicted deaths, and vertical lines indicate the start of the take−home policy.

Before the policy change, the slopes of methadone-involved deaths were flat in Large Central Metro, Medium Metro, and Noncore areas or declining in Large Fringe Metro, Small Metro, and Micropolitan areas. See Table 2 and Fig. 1. In April 2020, an abrupt shift occurred. Methadone-involved deaths increased sharply in Large Central Metro counties (45.84 deaths, 95 % CI, 25.65–66.04; p < .001), Large Fringe Metro (27.88 deaths, 95 % CI, 17.02–38.75; p < .001), Medium Metro (22.00 deaths, 95 % CI, 10.67–33.33; p < .001), Small Metro (9.41 deaths, 95 % CI, 5.00–13.82; p < .001), and Micropolitan counties (4.43 deaths, 95 % CI, 0.55–8.31; p = .026). The increase was not significant in Noncore areas.

**Table 2:** Interrupted time series analysis estimates for monthly overdose deaths involving methadone, January 2018 – June 2022, by urban-rural categories⁎.

**Parameter**	**Estimate**	**(95 % CI) P-value**	
**Large Central Metro:**			
Monthly trend (slope) before the take-home policy change	0.03	(–0.69–0.75)	.931
Change in number of overdose deaths at time of policy change	45.84	(25.65–66.04)	< .001
Monthly trend (slope) after the policy change	–1.36	(–2.32 to –0.40)	.007
Difference between slopes (before *minus* after)	–1.39	(–2.60 to –0.18)	.025
**Large Fringe Metro:**			
Monthly trend (slope) before the take-home policy change	–0.53	(–0.98 to –0.07)	.024
Change in number of overdose deaths at time of policy change	27.88	(17.02–38.75)	< .001
Monthly trend (slope) after the policy change	–0.62	(–1.07 to –0.17)	.008
Difference between slopes (before *minus* after)	–0.10	(–0.74–0.55)	.766
**Medium Metro:**			
Monthly trend (slope) before the take-home policy change	–0.29	(–0.70–0.12)	.162
Change in number of overdose deaths at time of policy change	22.00	(10.67–33.33)	< .001
Monthly trend (slope) after the policy change	–0.45	(–1.01–0.10)	.108
Difference between slopes (before *minus* after)	–0.16	(–0.91–0.58)	.665
**Small Metro:**			
Monthly trend (slope) before the take-home policy change	–0.27	(–0.44 to –0.10)	.003
Change in number of overdose deaths at time of policy change	9.41	(5.00–13.82)	< .001
Monthly trend (slope) after the policy change	–0.20	(–0.40–0.002)	.052
Difference between slopes (before *minus* after)	0.07	(–0.20–0.34)	.623
**Micropolitan (Nonmetro):**			
Monthly trend (slope) before the take-home policy change	–0.27	(–0.44 to –0.11)	.002
Change in number of overdose deaths at time of policy change	4.43	(0.55–8.31)	.026
Monthly trend (slope) after the policy change	0.06	(–0.15–0.27)	.577
Difference between slopes (before *minus* after)	0.33	(0.06–0.60)	.017
**Noncore (Nonmetro):**			
Monthly trend (slope) before the take-home policy change	–0.04	(–0.21–0.14)	.682
Change in number of overdose deaths at time of policy change	2.18	(–0.70–5.06)	.135
Monthly trend (slope) after the policy change	–0.04	(–0.19–0.10)	.547
Difference between slopes (before *minus* after)	–0.01	(–0.23–0.22)	.940

^⁎^ Note: Categories based on the 2013 National Center for Health Statistics urban-rural county classification scheme.

After the policy change, Large Central Metro counties saw a significant reduction in the slope of monthly methadone-involved deaths (–1.39 deaths, 95 % CI –2.60 to –0.18; p = .025) relative to their pre-intervention slope. In contrast, Micropolitan counties experienced a significant increase in their post-intervention slope (0.33 deaths, 95 % CI = 0.06–0.60; p = .017). Large Fringe Metro, Medium Metro, Small Metro, and Noncore counties did not have significant changes in slope.

### 3.2 Methadone deaths stratified by co-involvement of synthetic opioids (mostly fentanyl)

Table 3 presents the results of the fentanyl stratification analysis. In Large Central Metro areas, methadone deaths with fentanyl co-involvement showed a non-significant decrease in slope (–0.75, 95 % CI –1.62–0.11; p = .085), while a significant decrease was observed without fentanyl (–0.64, 95 % CI –1.24 to –0.03; p = .040). In the Large Fringe, Medium, and Small Metro categories, slope changes were not statistically significant, either with or without fentanyl presence. In Micropolitan (Nonmetro) areas, the slope increased with fentanyl involvement but did not reach significance (0.16, 95 % CI –0.01–0.32; p = .070); without fentanyl, the increase was significant (0.18, 95 % CI 0.02–0.34; p = .026). In Noncore (Nonmetro), fentanyl presence was associated with a slope increase (0.05, 95 % CI 0.01–0.09; p = .022), whereas its absence showed no significant change (–0.06, 95 % CI –0.25–0.13; p = .544).

**Table 3:** Interrupted time series analysis estimates of the difference between slopes in monthly methadone-involved overdose deaths before and after the take-home policy change, stratified by fentanyl co-involvement, January 2018 – June 2022⁎.

**Parameter**	**Estimate**	**(95 % CI)**		**P − value**
**Large Central Metro:**				
Before/After difference in slopes – with fentanyl	−0.75	(−1.62 to 0.11)		.085
Before/After difference in slopes – without fentanyl	−0.64	(−1.24 to –0.03)		.040
**Large Fringe Metro:**				
Before/After difference in slopes – with fentanyl	0.09	(−0.27 to 0.45)		.615
Before/After difference in slopes – without fentanyl	–0.19	(−0.66 to 0.28)		.427
**Medium Metro:**				
Before/After difference in slopes – with fentanyl	−0.07	(−0.44 to 0.29)		.687
Before/After difference in slopes – without fentanyl	−0.09	(−0.55 to 0.37)		.703
**Small Metro:**				
Before/After difference in slopes – with fentanyl	–0.002	(−0.10 to 0.10)		.961
Before/After difference in slopes – without fentanyl	0.06	(−0.19 to 0.32)		.614
**Micropolitan (Nonmetro):**				
Before/After difference in slopes – with fentanyl	0.16	(–0.01–0.32)		.070
Before/After difference in slopes – without fentanyl	0.18	(0.02–0.34)		.026
**Noncore (Nonmetro):**				
Before/After difference in slopes – with fentanyl	0.05	(0.01–0.09)		.022
Before/After difference in slopes – without fentanyl	−0.06	(−0.25 to 0.13)		.544

NOTES:

⁎ Categories based on the 2013 National Center for Health Statistics urban-rural county classification scheme.

### 3.3 Secular trend analysis

The analysis of secular trends showed that during the pre-intervention period there was no correlation between non-methadone overdose deaths and methadone-involved overdose deaths in any urbanization category (all p-values > 0.12), indicating that non-methadone deaths is not an appropriate comparator.

## 4 Discussion

This study examined the association between expanded access to take-home methadone doses and methadone-involved overdose deaths across urban and rural areas. Following the policy change, methadone-involved deaths declined significantly in Large Central Metro areas, remained unchanged in Large Fringe, Medium and Small Metro, and Noncore counties, and increased significantly in Micropolitan counties. This increase was not localized; our review of post-intervention Micropolitan deaths found methadone-related fatalities were dispersed across 44 states.

Because small, steady increases in deaths can result in progressively greater cumulative mortality over time, even a modest upward shift in mortality trends may signal important changes in population health outcomes, especially when reversing a prior decline. As shown in Fig. 1, although the magnitude of change may seem minor in Micropolitan areas, it nonetheless marks a break from the previous downward trend, raising concerns about the effectiveness of the policy change in Micropolitan areas.

Our stratified analysis showed only a limited association between fentanyl and changes in slope, nothwithstanding the proportionate rise in methadone deaths with fentanyl co-involvement across all urbanization categories (Table 1 and Figure S2). Although methadone/fentanyl deaths became more common over time, this alone does not imply a significant change in the trajectory of methadone/fentanyl fatalities following the policy change. Our analysis tested whether the rate of increase in these deaths, measured as deaths per month, significantly accelerated or decelerated after the policy change. A significant increase in slope was observed in Noncore counties, and an increase that approached significance in Micropolitan counties (p = .07; Table 3). Given their lower baseline rates of fentanyl co-involvement, this pattern may reflect structural vulnerabilities specific to rural regions. Limited access to harm reduction services and naloxone, for instance, may have amplified the risks when methadone was combined with illicit fentanyl use. In contrast, urban areas may have been buffered from increases in mortality despite rising co-involvement, potentially due to protective factors such as higher treatment retention, more robust overdose response systems, and a greater capacity to manage risks (Peters et al., 2020, Swann et al., 2021, Pasman et al., 2022).

It is important to note that basic data on OTP implementation of the take-home policy (e.g., the number of individuals who received take-home doses before and after the policy change) has not been systematically collected or reported (Taylor et al., 2023). This data gap prevents us from building a quantitative model of how OTP practices mediated the influence of urbanization on methadone-related deaths. However, consideration of the unique features of different communities may yield insights into the urban-rural disparities, though several interpretations offered here must remain speculative in the absence of more detailed data.

In Large Central Metro areas, the decline in methadone-related mortality may be partially attributed to a higher proportion of Black and Hispanic residents (Table 1). Prior research has shown that the take-home policy may have particularly benefited Black and Hispanic men (Harris et al., 2023), as daily OTP reporting can be a distressing and demeaning experience for marginalized individuals continually exposed to surveillance, stigma, and control (Frank et al., 2021, Peterkin et al., 2022, Abidogun et al., 2023). In addition to racial and ethnic disparities, sex-related factors may have contributed to overdose trends (D'Orsogna et al., 2023, Sauda et al., 2025, McCabe, 2024, Saraiya et al., 2024). While we did not stratify our analysis by sex, epidemiological data suggest that men generally experience higher overall overdose rates relative to drug misuse levels (Butelman et al., 2023), though women's usage rates may be higher for prescription opioids (Serdarevic et al., 2019). Differences in the challenges posed by the COVID-19 pandemic—such as increased caregiving responsibilities for women—also could have influenced patterns of methadone access and use. Future research should examine how sex-specific barriers impacted methadone treatment outcomes and overdose risk.

In contrast to the Large Central Metro areas, many rural communities saw increases in methadone-related overdose deaths, possibly due to COVID-19 disruptions, limited access to healthcare and harm reduction services, and strong views against drug use and treatment-seeking (Broman et al., 2023, Ezell et al., 2021). While rural communities vary, on the whole they may place a greater cultural emphasis on self-reliance, perceiving individuals struggling with addiction as weak or morally flawed, making it difficult for them to remain in treatment (Richard et al., 2020, Beachler et al., 2021, Brown et al., 2023). If, as seems likely, the take-home policy was not fully implemented in many locales, owing to concerns such as how long this option would last, perceived legal liability exposure for OTPs, and reduced reimbursements from fewer clinic visits (Wyatt et al., 2022, Meyerson et al., 2023), it may have been implemented less in conservative rural areas. For example, clinically stable patients may have received just a few additional take-home doses while less clinically stable patients received none (Levander et al., 2021), potentially limiting the benefits of the policy change. We could find no supporting evidence in the literature for the conjecture that rural methadone patients face more challenges with take-homes than urban patients due to being less clinically stable.

An alternative explanation for these trend line changes warrant consideration. To place the slope changes in practical context, it is useful to examine the number of deaths before and after the policy change (Table 1), while recognizing that the observed associations may reflect influences beyond the intervention itself. In April 2020, there was an abrupt increase in methadone-involved deaths across all urbanization categories, although the increase did not reach statistical significance in Noncore areas. Similar step increases occurred in drug overdose deaths not involving methadone (Stringfellow et al., 2023, Friedman and Akre, 2021) and in related domains such as alcohol-related deaths (Yeo et al., 2022, Case and Deaton, 2023). Disruptions related to COVID-19 are the probable cause of these increases rather than the change in the take-home methadone policy (Harris et al., 2023, U.S. Department of Health and Human Services, 2024, Harris, 2024). It was at this point in early spring 2020, with methadone deaths at record levels, that the trajectory in methadone deaths in Large Central Metro counties changed, beginning a steady decline. Could the methadone mortality declines in large urban areas simply reflect regression to the mean, the tendency for extreme values (the sharp increase in spring 2020) to be quickly followed by values closer to the mean (Davis, 1976)? Since the monthly decline in urban methadone-involved deaths unfolded gradually over a 27-month period, the trend is unlikely the result of a short-term random process but rather a shift in the conditions of methadone treatment in urban settings.

The overall increases in methadone-involved deaths observed in Table 1 following the March 2020 policy change largely reflect the abrupt spike that occurred in April 2020 and the months immediately after. As shown in Fig. 1 and detailed in Table 2, Table 3, while initial death counts rose, the subsequent trend in many areas was a decline in methadone-involved mortality. Most areas (Large Central, Large Fringe, Medium, and Small Metro) saw methadone mortality trending down after an initial spike, whereas Micropolitan areas continued to increase through 2022 and Noncore areas exhibited a generally flat slope in the unstratified analysis before and after the policy change, suggesting a different pattern of risk and response in rural settings.

A deeper, qualitative understanding of drug use, the social context of treatment, and OTP practices in rural and urban settings is necessary to assess these explanations. We especially need knowledge of patients’ lived experience to recognize and address the structural and cultural barriers faced by methadone-maintained patients in different communities (Adams et al., 2023).

Finally, it is useful to compare our study with that of Jones et al. (2022), as both use interrupted time series analysis to model monthly methadone-involved deaths. A critical difference lies in the measurement of methadone-involved deaths: Jones et al. chose a relative measure, calculating methadone-involved deaths as a *percentage* of non-methadone overdose deaths, but without offering an empirical justification for this metric. (Also, Jones et al. did not examine differences between rural and urban communities.) In contrast, we modeled the *absolute* number of monthly methadone-involved deaths. Our choice was supported by a secular trend analysis that found no statistically significant correlation between methadone and non-methadone overdose deaths prior to the policy change, which made it inappropriate to use non-methadone deaths as a denominator. Our re-analysis of Jones et al.'s time series data also found no correlation between methadone and non-methadone overdose mortality in the pre-intervention period (Spearman's r = 0.08, p = 0.79). Jones et al.'s measurement approach, not having an empirical basis, may have distorted the observed pre- and post-trends, potentially affecting the interpretation of their results.

### 4.1 Limitations

This study has limitations. First, the OTP take-home policy change occurred amid concurrent trends (e.g., COVID-19-induced unemployment and social isolation), economic policy changes (e.g., extended unemployment compensation, economic stimulus checks, eviction moratoriums) (Mulligan, 2022), and other policy changes (e.g., increased use of telemedicine) that could have influenced drug use and treatment for people with opioid use disorder. Second, the absence of a suitable control group – a sizeable number of persons taking methadone for opioid use disorder who were not exposed to the policy change – precludes our ability to draw causal inferences from the study data (Baicker and Svoronos, 2019), as information about non-exposed persons is currently insufficient (Roy et al., 2024, The Pew Charitable Trusts, 2022).^8,45^ Third, this study could not determine whether those who died from methadone-involved overdoses received the methadone through OTPs (about 90 % of all methadone supplies in the US are distributed to OTPs), from pharmacy dispensed prescriptions for pain (about 9 %), or from other sources, including diverted methadone (U.S. Department of Justice, Drug Enforcement Administration, 2018–2022). Of note, Kaufman et al. (2023) cross-state analysis of methadone supplies found no association between the distribution of methadone for pain and methadone overdoses, allaying concerns about misuse of methadone pain prescriptions. Fourth, the lack of systematic data on OTP implementation of the take-home policy—such as the number of individuals receiving take-home doses before and after the policy change—prevents a more precise assessment of its impact. Fifth, the 2013 NCHS Urban-Rural Classification Scheme predates the start of our study period (January 2018) and does not account for subsequent population shifts. The 2023 update reclassified about 2.5 % of counties. The 'true' classification likely falls between the two. Any deviation of the 2013 classification from this 'true' classification introduces some measurement error, though it is unlikely to materially affect our findings. Sixth, approximately 5 % of death certificates did not list the drugs involved in the overdose. Lastly, the 2022 provisional mortality data may minimally underestimate overdose deaths due to delayed reporting (Rossen et al., 2018).

### 4.2 Conclusions

This study found that the association between the take-home policy and methadone-involved deaths varied across rural and urban settings. Large Central, Large Fringe, Medium, and Small Metro counties experienced a sharp rise in methadone-involved deaths in April 2020, followed by declines. Micropolitan counties also experienced an initial spike, but unlike metro areas, deaths did not return to earlier levels. Noncore counties, which had a smaller uptick, saw a sustained increase in deaths involving both methadone and fentanyl.

The study results suggest the need to add new flexibilities and supports to enhance rural access and manage risks. This might entail broad expansion of mobile methadone units, audio-only counseling, and offsite drug testing in rural areas (Suen et al., 2023, Khazanov et al., 2023). The role of technology might also be expanded, using tools such as automated home medication dispensers and tele-monitoring systems (with broadband support) for regular check-ins (Sigmon et al., 2023). Additionally, the regulatory framework granting OTP directors control over methadone treatment and dispensing should be amended to permit addiction specialists to prescribe methadone and community pharmacies to dispense it (Amram et al., 2023, Conway et al., 2023, Wu et al., 2023, Modernizing Opioid Treatment Access Act, 2023). With the right incentives, local pharmacies could play a major role in improving the methadone treatment infrastructure. They might serve as sites for observed daily dosing and take-home methadone distribution, with the goals of lessening travel burdens, enhancing treatment accessibility and adherence, minimizing diversion, and reducing overdoses (Joudrey et al., 2020). These and similar initiatives will provide underserved communities with much-needed methadone treatment availability and patient-centered supports.

## CRediT authorship contribution statement

**Mandell David S:** Writing – review & editing, Supervision. **Long Judith A:** Writing – review & editing, Supervision. **Harris Rebecca:** Writing – review & editing, Writing – original draft, Methodology, Formal analysis, Data curation, Conceptualization. **Kranzler Henry R:** Writing – review & editing. **Bao Yuhua:** Writing – review & editing. **Perone Jeanmarie:** Writing – review & editing.

## Author disclosure

Dr. Kranzler is a member of advisory boards for Altimmune, Clearmind Medicine, Dicerna Pharmaceuticals, Enthion Pharmaceuticals, Lilly Pharmaceuticals, and Sophrosyne Pharmaceuticals; a consultant to Sobrera Pharmaceuticals and Altimmune; the recipient of research funding and medication supplies for an investigator-initiated study from Alkermes; a member of the American Society of Clinical Psychopharmacology's Alcohol Clinical Trials Initiative, which was supported in the last three years by Alkermes, Dicerna, Ethypharm, Imbrium, Indivior, Kinnov, Lilly, Otsuka, and Pear; and a holder of U.S. patent 10,900,082 titled “Genotype-guided dosing of opioid agonists,” issued 26 January 2021.

### Funding

Dr. Harris' research is supported by the 10.13039/100000026National Institute on Drug Abuse (K23 DA054147). Dr. Kranzler's involvement in this work is supported by the VISN 4 MIRECC, Crescenz VAMC.

## Declaration of Competing Interest

The authors declare that they have no known competing financial interests or personal relationships that could have appeared to influence the work reported in this paper.
