# Supplementary material for: Methadone-involved overdose deaths in urban and rural communities before and after the public health emergency flexibilities for methadone take-home doses
Source: Drug Alcohol Depend Rep. 2025 Apr 24;15:100339. doi: 10.1016/j.dadr.2025.100339 (PMC12127620; doi:10.1016/j.dadr.2025.100339)
Supplement: Supplementary file 1 — Supplementary material [file mmc1.docx]

**Supplement**

**Table of Contents:**

**Supplement Part A.** Interrupted time series analysis (ITSA) regression model

**Supplement Part B.** Secular trend analysis

**Supplement Figure S1.** Distribution of counties according to the National Center for Health Statistics Urban–Rural Classification Scheme for Counties.

**Supplement Figure S2.** Percent of methadone-involved deaths that also involved synthetic opioids (mostly fentanyl and analogs) by urban-rural categories.

**A. Interrupted time series analysis (ITSA) regression model**

The standard interrupted time series regression model takes the form:

*Y_t_* = *B*_0_ + *B*_1_*T_t_* + *B*_2_*X_t_*  + *B*_3_*X_t_T_t_* + e*_t_*

where

*Y_t_* is the outcome variable measured at each time point *_t_*

*T_t_* is the time since the start of the study

*X_t_* is a dummy variable representing the intervention (pre-intervention periods 0, otherwise 1)

*X_t_T_t_* is an interaction term

*B*_0_ is the intercept

*B*_1_ is the slope prior to the intervention

*B*_2_ is the change in the level of the outcome variable immediately following the introduction of the intervention (compared to the counterfactual)

*B*_3_ is the difference between the pre- and post-intervention slopes of the outcome, and

e*_t_* is the error term.

We used interrupted time series analysis to assess whether there was a change in the level or trend in monthly methadone-involved deaths following the SAMHSA take-home policy change. The pre-intervention period was January 2018 to March 2020. The post-intervention time period was April 2020 to June 2022. The model constructs a counterfactual – what the trend would look like in the absence of the policy change – which is then compared with the post-intervention actual trend.

**B.** **Secular trend analysis**

We examined whether non-methadone-involved overdose deaths could serve as a secular trend comparator. Non-methadone deaths met the two *a priori* criteria for a valid secular trend control: (1) a theory-based relationship with methadone deaths, as both may be influenced by broader social forces, despite differences in the affected populations, and (2) no direct relationship with the policy change, meaning trends in non-methadone deaths should remain unaffected by the methadone take-home policy. Additionally, we applied a third criterion, commonly used in time series intervention analyses for selecting a control group: assessing the pre-intervention association between the treatment and potential control group outcomes. A lack of association between the trendlines would indicate that non-methadone deaths are unsuitable as a comparator. This is because a valid comparator should exhibit similar pre-intervention trends to the treatment group, reflecting shared underlying factors influencing both series over time. Without such an association, we cannot assume that non-methadone deaths would serve as an appropriate counterfactual for methadone-involved deaths in the absence of the policy change.

We assessed the association between monthly methadone-involved and non-methadone-involved overdose deaths during the pre-intervention period (January 2018–March 2020) using Spearman’s rank-order correlation coefficient (*r_s_*). We used *r_s_* because it is sensitive to monotonic relationships between trends, regardless of whether the association is linear. The formula for *r_s_* is:

$$r_{s} = 1 - \frac{6\sum d\begin{matrix} 2 \\ i \end{matrix}}{n(n^{2}-1)}$$

where

*r_s_* is Spearman's rank-order correlation coefficient,

*d_i_* is the difference between the ranks of paired observations (methadone vs. non-methadone deaths in month *i*), and

*n* is the number of monthly observations in the pre-intervention period (January 2018–March 2020, or 27).

**Figure S1.** Distribution of counties according to the 2013 National Center for Health Statistics Urban–Rural Classification Scheme for Counties.

**
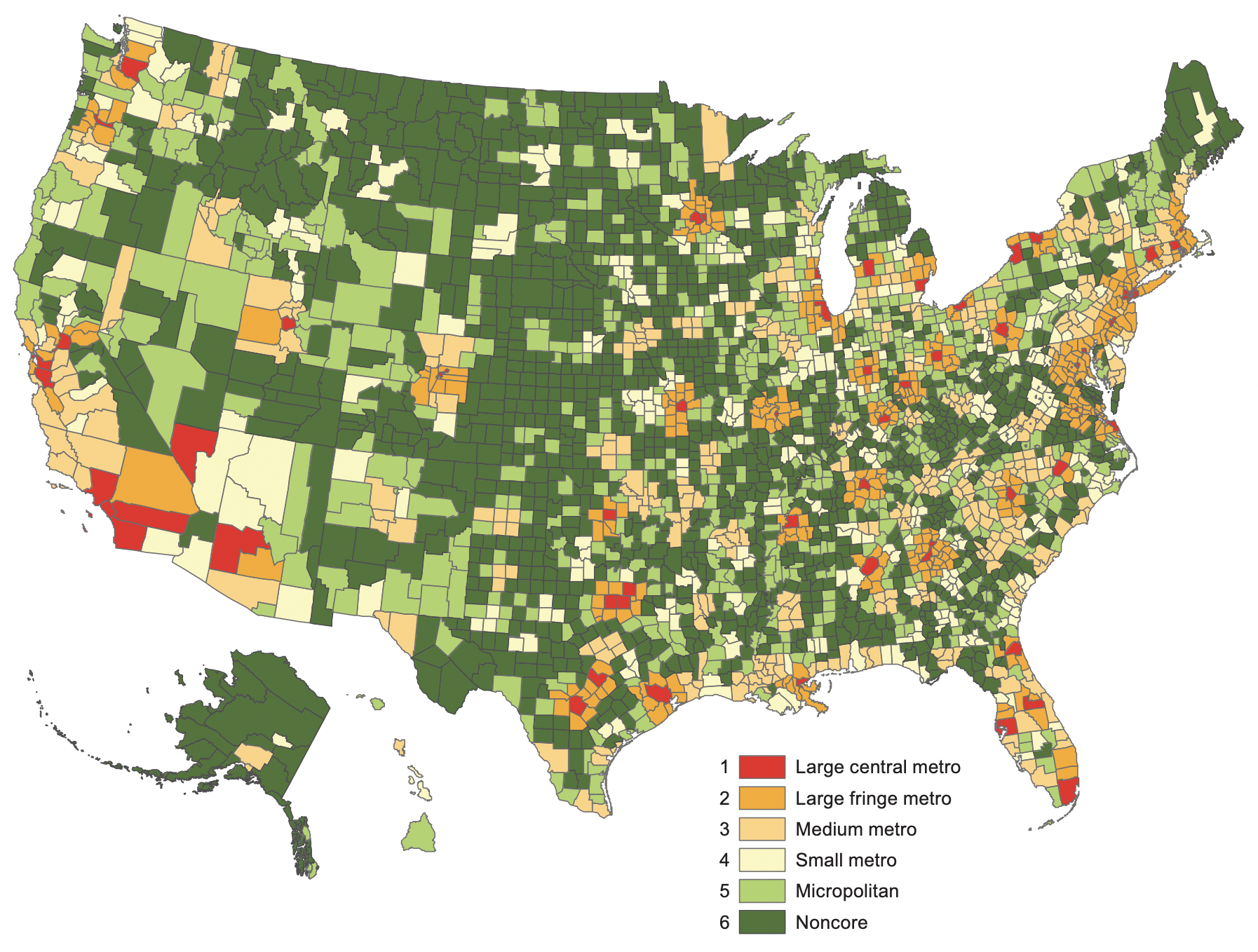
**

Source: Ingram DD, National Center for Health Statistics. Vital Health Stat. 2014; 2(166).

**Figure S2.** Percent of methadone-involved deaths that also involved synthetic opioids (mostly fentanyl and analogs) by urban-rural categories, January 2018 – June 2022.

SOURCE: Authors’ analysis of monthly overdose mortality data from the National Vital Statistics System, CDC WONDER online database.

NOTES: Categories based on the National Center for Health Statistics urban-rural county classification scheme. Data are LOWESS smoothed (running means).
